# Longitudinal trends in vaping, smoking, and harmful alcohol use across sexual orientations in the UK (2014–2021)

**DOI:** 10.1371/journal.pone.0339847

**Published:** 2026-01-16

**Authors:** Yihong Bai, Peiya Cao, Chungah Kim, Kristine Ienciu, Gwen Ehi, Antony Chum

**Affiliations:** 1 School of Kinesiology and Health Science, York University, Toronto, Ontario, Canada; 2 Department of Community Health Sciences, University of Manitoba, Winnipeg, Manitoba, Canada; National Institutes of Health, University of the Philippines Manila / De La Salle University, PHILIPPINES

## Abstract

**Background:**

Sexual minorities, including lesbian, gay, and bisexual individuals, often face higher substance use rates due to societal stressors. Recent societal changes, including the COVID-19 pandemic and rising hate crimes in the UK, may have impacted these disparities across sexual orientations. This study aims to examine changes in vaping, smoking, and alcohol use disparities across sexual orientations in the UK from 2014 to 2021.

**Method:**

Data from the UK Longitudinal Household Survey (2014–2021) were analyzed, covering 42,052 participants aged 16 + . Sexual orientation categories included heterosexual, gay/lesbian, bisexual, and other sexual minorities. The outcomes were smoking, vaping, and harmful alcohol drinking. Longitudinal logit models with generalized estimating equations were used, adjusting for demographic factors. Predictive prevalences were calculated and used to examine the disparities between each sexual minority group and the heterosexual group over time.

**Results:**

We did not find consistent increases in substance use disparities across sexual orientation groups in the UK from 2014 to 2021. Small differences were observed among bisexual individuals who had a higher predicted prevalence of harmful alcohol drinking in 2019–2020 (~11–13% higher), while gay/lesbian individuals showed higher vaping prevalence in 2019 (~5% higher) than heterosexual individuals. For smoking, no disparities were observed.

**Conclusion:**

Unlike prior studies that reported disparities, our longitudinal analysis found little to no evidence that sexual minorities had elevated or widening substance use disparities. While some subgroups exhibited elevated risks in specific years, these patterns were not sustained over time. Future research should explore how social stressors, policy contexts, and community norms interact to inform targeted, inclusive interventions that reflect the diversity within LGBTQ+ populations.

## Introduction

The aim of this paper is to investigate changes in disparities in vaping, smoking, and alcohol use across sexual orientations in the UK from 2014–2021. Smoking remains a leading cause of preventable illness and death, contributing to lung cancer, heart disease, and stroke [1]. Vaping has emerged as a popular alternative, with recent evidence suggesting that vaping may pose serious acute respiratory health risks [2]. While some argue that it may offer a safer alternative to smoking, the long-term health effects of vaping are still largely unknown [3]. Similarly, individuals who engage in harmful alcohol consumption are at a significantly increased risk of developing alcohol use disorder, which is a leading cause of death and disability, linked to liver disease, cancer, and mental health issues [4]. Given that these substances are widely available and subject to regulations, understanding their consumption patterns can provide valuable insights into the effectiveness of existing policies.

A substantial body of research has documented that individuals who identify as sexual minorities, including those who describe themselves as lesbian, gay, or bisexual, exhibit higher rates of substance use disorders when compared to their heterosexual counterparts [5–8]. These disparities may be attributable, at least in part, to the social stress that sexual minority individuals disproportionately experience as a result of societal stigma, discrimination, and other minority stressors [8]. These disparities can be theoretically understood through the lens of minority stress theory, which argues that sexual minorities experience elevated levels of psychological distress and subsequent health-risk behaviors, such as substance use, due to unique stressors linked to societal stigma, discrimination, and marginalization [9–11].

Recent societal stressors in the UK have intensified, particularly following the substantial rise in hate crimes against sexual minorities after 2017, with recorded offences based on sexual orientation increasing from 8,569 in 2016/17–17,135 in 2020/21—approximately a 100% increase over this period [12]. In a 2023 parliamentary debate, Members of Parliament highlighted a 217% increase in anti-LGBTQ+ hate crimes since 2017–18 [13], drawing attention to this escalation during the study period and fueling extensive public discourse. Local responses also drew national attention—for instance, in June 2021, a string of homophobic assaults in Liverpool prompted a public protest supported by the city’s mayor and police commissioner, bringing urgent media and civic attention to LGB-targeted violence [14]. The convergence of these political and public dynamics likely intensified minority stress among LGB individuals, reinforcing conditions that may contribute to health-risk behaviors. Moreover, while the COVID-19 pandemic introduced widespread psychosocial challenges, sexual minorities might have experienced a disproportionately negative impact due to disruptions in critical social support networks, heightened isolation, and pre-existing inequities in mental health and healthcare access [15,16]. For example, a UK-based study found that COVID-19 disproportionately affected the mental health of sexual minority individuals [11]. Psychological distress was assessed using the 12-item General Health Questionnaire, which captures symptoms such as anxiety, depressed mood, and social dysfunction. Results showed that lesbian women experienced significantly greater increases in distress compared with heterosexual women, indicating heightened risks of depression and anxiety symptoms. Furthermore, the closure of LGBTQ+ community centers and reduced physical contact with chosen families can create distinct challenges [17], potentially diminishing feelings of belonging and affirmation. Research has also highlighted the challenges faced by LGBTQ+ individuals quarantined with homophobic or transphobic family members [18]. Consequently, we hypothesize that these societal events (post-2017 rise in hate crimes and the COVID-19 pandemic) may have intensified substance use disparities between sexual minorities and heterosexual individuals over time. Sexual minorities may turn to substance use as a coping mechanism in response to these stressful experiences [8,19,20]. Monitoring the longitudinal trends in substance use is crucial to ascertain whether there may be growing health disparities that necessitate targeted interventions.

### Prior literature on alcohol, smoking, and vaping among LGB people

Prior research consistently indicates that LGB+ individuals face higher rates of substance use, particularly alcohol misuse, compared to their heterosexual counterparts. In the UK, numerous studies have found this elevated risk in sexual minorities [7,21]. Qualitative findings suggest that alcohol is often used both as a coping mechanism for stress and discrimination and within LGBTQ+ social contexts [21]. A UK study using the (2014–2016) UK household survey reported higher hazardous alcohol use among lesbian/gay (25%) and bisexual (31%) individuals compared to heterosexual individuals (13%), even after adjusting for demographics [22]. Furthermore, in the U.S., cross-sectional data indicate that gay and bisexual men show the highest alcohol use disorder rates from ages 18–45, while lesbian and bisexual women are more affected later in life [23]. Among LGB youth in the U.S., particularly boys and young men, alcohol use began earlier and occurred more frequently than in their heterosexual peers, with these elevated levels persisting into adulthood [24].

Studies in the US and Canada (1996–2020) showed persistent tobacco use disparities in LGB groups, although disparities in heavy use among youth have decreased over time [25]. However, evidence on LGB adults in these countries is limited. Meanwhile, in the UK, a (2014–2016) study found higher tobacco use among LGB individuals compared to heterosexuals, though these differences were largely explained by sociodemographic factors [22]. A longitudinal UK study (1991–2017) found persistent smoking disparities between sexual minorities and heterosexual individuals, with particularly pronounced differences among sexual minority women, despite overall declines in smoking rates [26]. However, this study did not differentiate between bisexual and homosexual individuals, likely due to sample size. Furthermore, another UK study (2013–2019) found modest declines in smoking among heterosexuals, stable rates among bisexuals, and sharp drops among gay and lesbian individuals, though it lacked sex/gender-specific analysis [27]. To better understand these trends, future studies should disaggregate by sexual orientation (e.g., separating bisexual individuals—who face distinct stereotypes and stressors from gay and lesbian individuals [28], as well as by gender (to capture potential differences in experiences and stress processes between gay men and lesbians [29] and age groups [29], in order to reveal more detailed and nuanced patterns.

Despite being an emerging topic, research on vaping disparities related to sexual orientation is limited and mainly US-focused. A recent cross-sectional U.S. study (2020−21) found higher odds of vaping in gay/lesbian youth (but not bisexual) even after accounting for sex and ethnoracial identity [30]. Another U.S. study using a non-random sample focusing on smoking/vaping trajectories revealed distinct patterns in vaping: among men, usage was highest among bisexual individuals, followed by heterosexual and then gay men; among women, bisexual individuals also had the highest usage, followed by lesbians and then heterosexual women [31]. However, a 2019 U.S. study using a non-random panel found no significant differences in current vaping between sexual minority and heterosexual adolescents, though the sample size was smaller and the sex/ sexual orientation categories were not disaggregated [32]. As well, another UK national cross-sectional study (2013−19) also found no significant differences in vaping between heterosexual individuals and gay/lesbian, bisexual, or other individuals [27].

Despite growing interest in substance use disparities across sexual orientations, existing research faces several key limitations. Much of the literature relies on cross-sectional data, limiting the ability to track changes over time [ [22,23,27,33,34]. The literature primarily focuses on youth populations, which limits its generalizability [24,35–37]. Moreover, there is a lack of longitudinal studies, and many studies draw from a non-representative sample [7,31,35,36]. Many studies also group lesbian, gay, and bisexual individuals, which can obscure important differences between subgroups [26,33. Additionally, most longitudinal research is U.S.-centric [26]. As well, it often relies on data collected between the 1990s and mid-2010s, and rarely includes other sexual minority identities such as queer, asexual, and pansexual individuals [7,23,26,35,37,38]. Recognizing these prior limitations, we propose the following research question using a nationally representative sample: To what extent have disparities in vaping, smoking, and harmful alcohol drinking changed across sexual orientations (i.e., gay/lesbian, bisexual, heterosexual, and other sexual minorities) in the UK from 2014 to 2021?

## Materials and methods

### Data

We used data from the UK Longitudinal Household Survey (UKHLS), also known as ‘Understanding Society’, a nationally representative study that began in 2009. This longitudinal survey annually engages participants throughout the UK, with over 50,000 participants across more than 35,000 households. Details regarding the sampling framework, data collection, and yearly response rates are accessible through the Understanding Society website [39]. UKHLS data are fully anonymized and publicly accessible and can be downloaded by registering on the UK Data Service website (https://ukdataservice.ac.uk/). The smoking variables were collected from waves 6–13, the drinking variables from waves 7, 9, 11–13, and the vaping variable from waves 8–13. The scope of the present analysis is limited to 2014–2021, spanning waves 6–13. We used an unbalanced panel sample, which means that individuals were included in the analysis even if they did not participate in all waves. On average, participants contributed data for 5.6 waves, with similar retention rates across groups (Heterosexual: 5.65; Gay/Lesbian: 5.72; Bisexual: 5.36; Other: 5.45). A detailed breakdown of sample size by wave and sexual orientation is provided in Appendix Table S1 in S1 File. 2022 and 2023 were excluded due to limited observations. Individuals aged 16 or older in the baseline year were included, as sexual orientation was only asked among respondents aged 16 or above in the UKHLS.

### Measures

#### Sexual orientations.

The sexual orientation of participants was determined through a single question: “Which of the following options best describes how you think of yourself?” Available responses included “heterosexual or straight,” “gay or lesbian,” “bisexual,” “other” (defined as non-heterosexual, non-LGB), and “prefer not to say.” The category labelled ‘other’ encompasses individuals who do not identify as heterosexual, gay, or bisexual and may include those identifying as queer, pansexual, questioning, or asexual. We excluded respondents who selected “prefer not to say,” as this group (approximately 2.5% of respondents) likely represents a diverse array of backgrounds, including individuals reluctant to disclose their sexual orientation, those with privacy concerns, those morally opposed to the question, and those who may not understand the question. Data on sexual orientation were provided exclusively by adults who consented to and participated in the UKHLS self-completion interview. The reference category for comparative analyses was “heterosexual or straight.

#### Outcomes.

The variable representing smoking status was constructed from the response to the query, “Do you smoke cigarettes?” This variable was coded as 1 for current smokers and 0 for non-smokers. The variable pertaining to the use of electronic cigarettes (vaping) was obtained from the response to the question, “Do you use electronic cigarettes (e-cigarettes)?” Respondents could choose from six options, ranging from never having used e-cigarettes to using them at least once a week. The vaping variable was dichotomized, assigned a value of 1 for respondents who use e-cigarettes at least once a month, and 0 otherwise, following prior studies [40,41]. Harmful alcohol drinking was assessed using the Alcohol Use Disorders Identification Test (AUDIT-C), which consists of three specific questions about frequency of alcohol use, typical quantity consumed, and frequency of heavy episodic drinking, detailed in supplementary Table S2 in S1 File. The AUDIT-C is scored on a scale from 0 to 12, with higher scores indicating a greater likelihood of alcohol consumption impacting health and safety. According to the guidelines, the assessment of harmful alcohol drinking was also binary, with a threshold of 4 or more for males and 3 or more for females defining the presence of harmful alcohol drinking, and scores below these thresholds indicating its absence [42].

#### Covariates.

Covariates for the study included ethnoracial status (coded as White: British/English/Scottish/Welsh/Northern Irish vs. all other ethnic backgrounds), rurality (urban vs. rural), education (post-secondary vs. no post-secondary), employment (employed vs. unemployed), total person net income (in thousands of pounds), self-reported sex, and age (in years). We also included the following fixed effects: region (12 administrative regions of the UK) and year of survey.

#### Statistical analysis.

To investigate the temporal trends in substance use disparities between heterosexual individuals and sexual minority groups throughout the study period, we utilized a population average approach employing logit regression, adjusting for all covariates for our binary outcomes. This methodology yields consistent and robust estimates of the average effect of predictors on the outcome across the entire population, as opposed to subject-specific effects [43]. Contrary to subject-specific models, which interpret effects within a random effects framework, the population average approach via generalized estimating equations (GEE) exhibits reduced sensitivity to assumptions about the within-panel error correlation structure [44]. This characteristic mitigates biases that may arise from misspecified random effects models [45]. Additionally, this approach adeptly handles unbalanced panels and varying time intervals between observations, thereby enhancing its suitability for observational studies where data collection may not be uniform [46]. The models incorporated variables for time (represented by dummy variables for each year), sexual orientations (represented by three dummy variables), and their interactions. These interaction terms allow us to estimate how the association between sexual orientation and each substance use outcome varies over time. From these models, we derived fully adjusted predicted probabilities for each sexual orientation group across years. This approach allows us to track changes in substance use prevalence within each group over time, before examining disparities between groups. Then, the disparities for each sexual orientation relative to heterosexual orientation were annually assessed by the marginal effect of sexual orientations over the years. In addition, we stratified the models by sex. Longitudinal weights corresponding to the self-completion interview were employed to account for the restricted sample and adjust for potential non-response bias. All analyses were conducted using STATA v18.

#### Sensitivity tests.

We conducted a series of sensitivity tests to assess the robustness of our findings. First, we stratified the models by age, partner status, and parenthood status (separately) to explore potential heterogeneous variations [47]. The age stratification was divided into two categories: individuals under 30 years of age and those aged 30 or above. Second, alternative outcome measures were employed to further validate our results, which included 1) the continuous AUDIT-C scores, 2) the continuous usual number of cigarettes smoked per day, and 3) a binary cut-off for a higher intensity of vaping (=1 if used at least once a week).

### Ethics statement

The paper uses secondary fully anonymized data from the “Understanding Society: The UK Household Longitudinal Study (UKHLS)”, and the authors did not have access to potentially identifying information; thus, obtaining participants’ informed consent is not applicable. The University of Essex was responsible for the UKHLS data collection/management, and has obtained verbal informed consent from all the study participants. Interviews were primarily conducted face-to-face in respondents’ homes by trained interviewers or completed by respondents themselves online. Each section of the questionnaire, including all questions, was answered voluntarily. Additional details about the UKHLS survey—including study design, sampling, timeline, questionnaire structure, interview process, fieldwork procedures, response rates, data collection, and data processing—are available at: https://www.understandingsociety.ac.uk/documentation/mainstage/user-guides/main-survey-user-guide/. No additional ethical approval was necessary for this project. The UKHLS datasets are available on the UK Data Service platform and the URL is as follows: https://beta.ukdataservice.ac.uk/datacatalogue/series/series?id=2000053#!/access-data.

## Results

**Fig 1:**
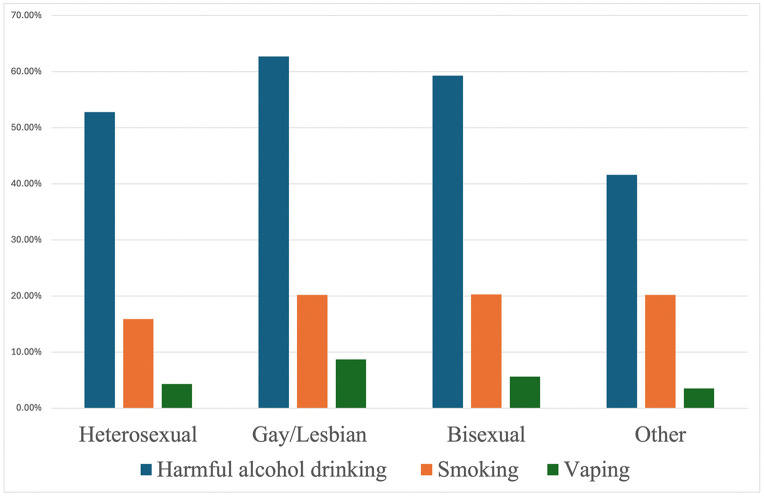
Prevalence of harmful alcohol drinking, smoking, and vaping across sexual orientations at baseline.

The baseline descriptive statistics of the study participants (n = 42,052) are detailed in Table 1. The baseline sample included 95.3% heterosexual individuals, 1.5% gay/lesbians, 1.9% bisexual individuals, and 1.3% people with other sexual orientations. The bisexual group was the youngest, least likely to be married, and had the lowest income among all the sexual orientations. Fig 1 presents the substance use prevalence at baseline: gay/lesbian people had the highest percentages with harmful alcohol drinking (62.7%) and vaping (8.7%), while the prevalence of smoking was around 20% among the gay/lesbian, bisexual people, and people with other sexual orientations, higher than the heterosexual group (16%).

**Table 1:** Baseline sample characteristics across sexual orientations (unweighted).

	Heterosexual	Gay/Lesbian	Bisexual	Other
N	40074	644	805	529
Men	45.1%	59.2%	35.9%	40.8%
Post-secondary education	36.3%	45.0%	29.8%	24.8%
Employed	56.4%	63.3%	47.9%	44.7%
Married	61.7%	41.7%	36.7%	52.9%
White: British/English /Scottish/Welsh /Northern Irish	79.4%	84.0%	76.1%	64.1%
Urban	75.5%	83.1%	79.7%	81.3%
Region				
North East	3.7%	3.9%	4.7%	2.5%
North West	10.2%	12.6%	8.6%	7.6%
Yorkshire and the Humber	8.4%	8.2%	8.1%	7.9%
East Midlands	7.1%	7.0%	8.1%	6.4%
West Midlands	8.3%	6.1%	8.1%	11.5%
East of England	8.4%	7.0%	7.0%	4.0%
London	12.0%	18.0%	14.8%	18.9%
South East	11.8%	11.3%	15.2%	11.2%
South West	8.1%	7.8%	8.2%	4.7%
Wales	6.9%	7.6%	6.5%	10.4%
Scotland	8.6%	5.6%	7.3%	7.6%
Northern Ireland	6.7%	5.0%	3.5%	7.4%
Age	46.55 (19.0)	38.42 (16.2)	31.60 (16.5)	44.36 (19.9)
Monthly income in thousands	1.49 (3.02)	1.56 (1.77)	1.01 (1.4)	1.06 (1.0)

**Fig 2:**
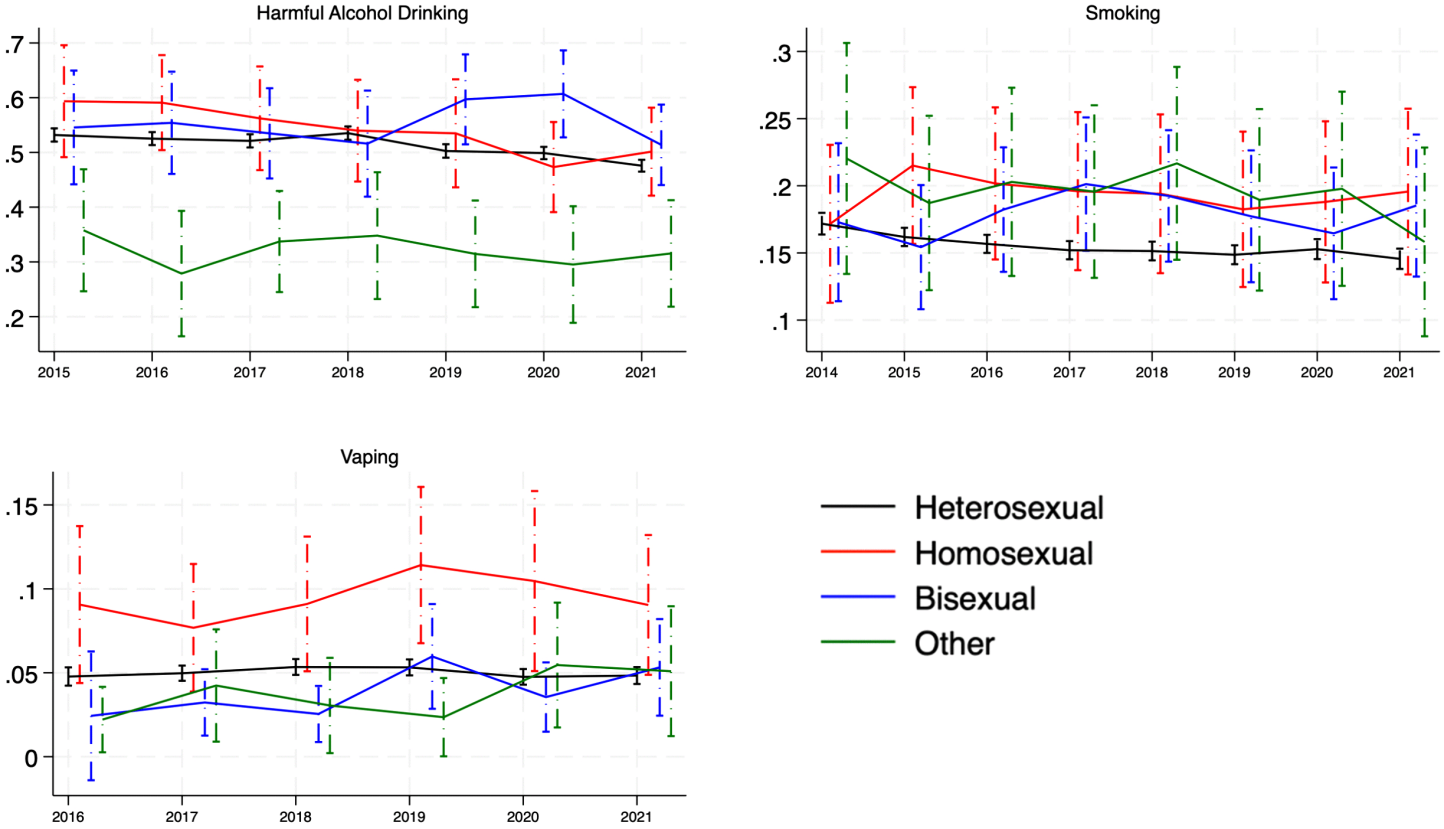
Predictive probabilities of harmful alcohol drinking, smoking, and vaping disparities across sexual orientations over time: fully adjusted probability estimates and 95% confidence intervals.

Fig 2 presents the predictive probabilities of our outcomes across sexual orientations over time. For harmful alcohol drinking, bisexual individuals showed higher predicted probabilities than other groups in 2019–2020, while the “other” group had lower probabilities throughout the period. In terms of smoking, differences across groups were generally small and fluctuated, with overlapping confidence intervals in most years. For vaping, gay/lesbian individuals exhibited a higher predicted prevalence beginning in 2018, peaking around 2020, whereas the “other” group showed consistently lower predicted probabilities compared to heterosexual individuals.

**Fig 3:**
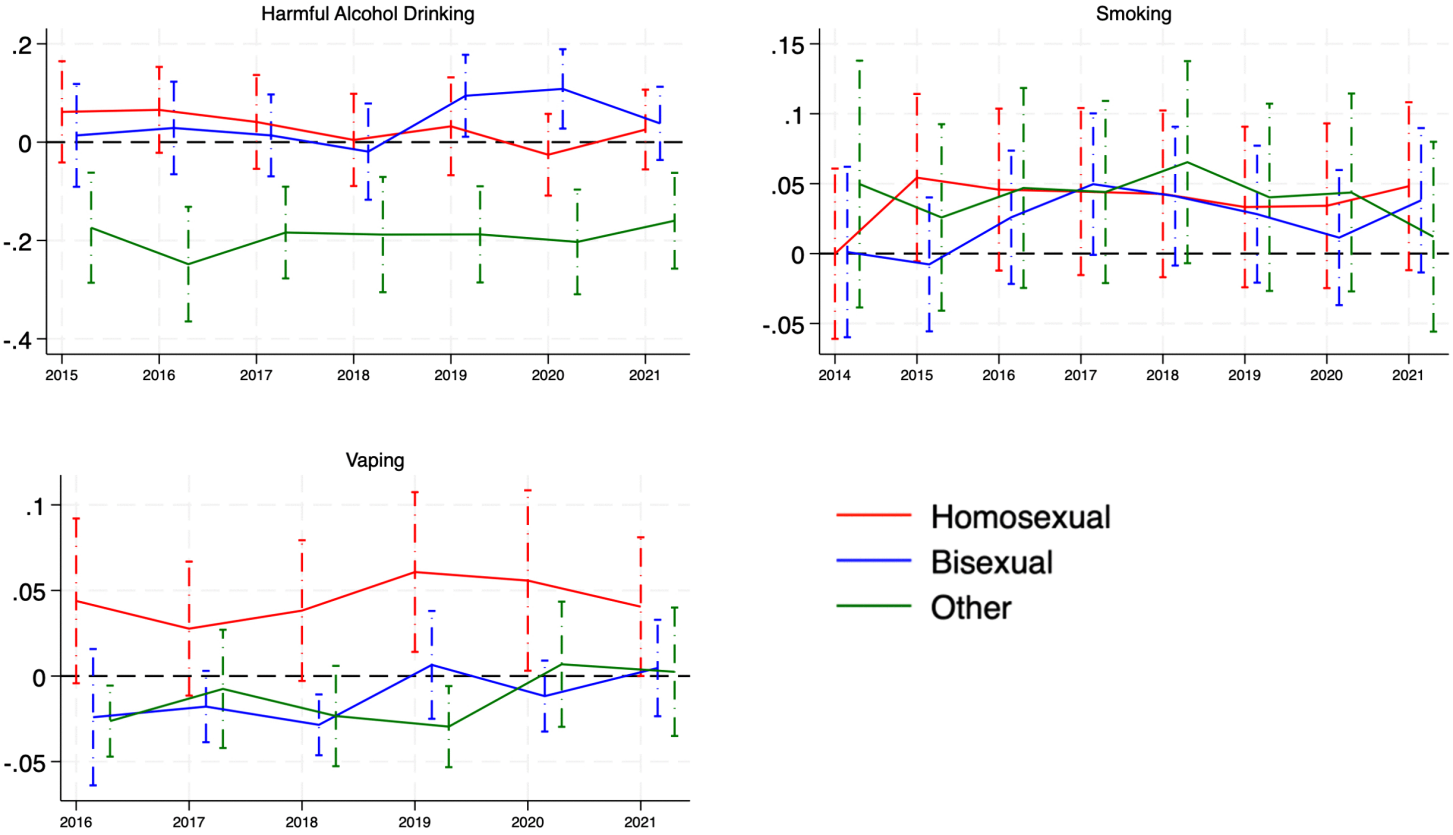
Longitudinal disparities of harmful alcohol drinking, smoking, and vaping disparities across sexual orientations versus heterosexual people: fully adjusted probability estimates and 95% confidence intervals for the difference between each non-heterosexual group vs the heterosexual group over time.

Our model results (Fig 3 and Tables S3-S5 in S1 File) showed that, in 2019, the predicted prevalence of harmful alcohol drinking for bisexual people was 11.4% (95% CI: 1.5% to 21.3%) higher than the heterosexual group; and in 2020, the disparity was 13.5% (95%CI: 4.4% to 22.6%). The predicted prevalence of harmful alcohol drinking for the ‘other’ group was consistently lower than the heterosexual group (ranging from 10% to 25%), while no differences were observed between gay/lesbian and heterosexual individuals over the study period. As for smoking, no differences were observed among gay/lesbian, bisexual, and ‘other’ groups versus the heterosexual group over the study period. For vaping, the predicted prevalence for gay/lesbian was 5.6% (95% CI: 0.7% to 10.5%) higher than the heterosexual group in 2019. The predicted prevalence of vaping for bisexual individuals was around 3.1% (95% CI: −5% to −1.1%) lower than for heterosexual individuals in 2018. Similarly, the predicted prevalence for the ‘other’ group in vaping was around 3–4% lower than their heterosexual counterparts in 2016, 2018, and 2019.

**Fig 4:**
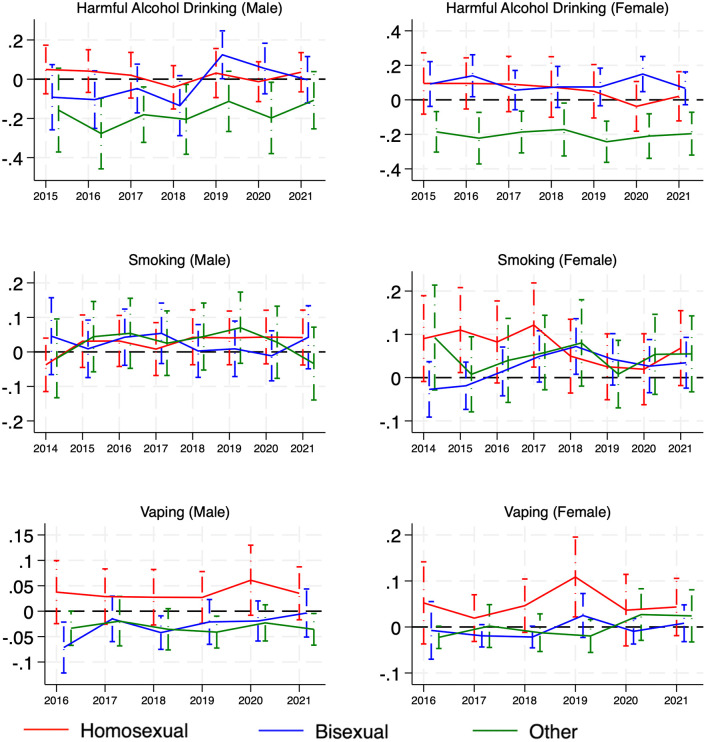
Longitudinal disparities (by sex) of harmful alcohol drinking, smoking, and vaping disparities across sexual orientations versus heterosexual people: adjusted probability estimates and 95% confidence intervals for the difference between each non-heterosexual group vs the heterosexual group over time.

When stratified by sex (Fig 4), the ‘other’ women group had consistently lower predicted prevalence in harmful alcohol drinking compared to their heterosexual counterparts over the study period, while the ‘other’ men group had a lower predicted prevalence in harmful alcohol drinking in four years (year 2016, 2018, 2020, 2021) out of the eight-year study period. For smoking, a higher predicted prevalence was observed for lesbian women in 2015 and 2017. Lesbian women also had higher levels of vaping compared to heterosexual women in 2019.

In the sensitivity analysis stratified by age groups (Figure S1 in S1 File), relative to heterosexual individuals aged 30 and above, stronger effects were observed for ‘other’ individuals in the older group (aged 30 and over) in harmful alcohol drinking. ‘Other’ individuals in the younger group had a lower predicted prevalence of smoking than their heterosexual counterparts in 2021. In 2021, gays/lesbians aged 30 and above showed a higher predicted prevalence of vaping than gays/lesbians under 30 relative to their heterosexual counterparts, while younger gays/lesbians (aged under 30) were observed lower predicted prevalence of vaping. In the partner status–stratified analysis (Figure S2 in S1 File), non-partnered gay/lesbian and bisexual individuals showed elevated predicted probabilities relative to non-partnered heterosexuals. Among partnered individuals, most differences were smaller and less consistent over time. In the parenthood-stratified analysis (Figure S3 in S1 File), larger disparities were observed among non-parents for smoking and harmful alcohol use, particularly among bisexual and “other” groups. Among parents, differences in vaping became more prominent in later years, especially for gay/lesbian individuals. Analyses using alternative outcomes (Figure S4 in S1 File) showed similar results to our main analyses.

## Discussion

This study did not find clear or consistent patterns of increasing substance use disparities across sexual orientation groups in the UK from 2014 to 2021. For most groups and outcomes, differences in predicted substance use prevalence compared to heterosexual individuals were small, statistically non-significant, and remained relatively stable over time. These findings do not support our hypothesis that disparities in substance use across sexual orientation groups widened during this period. One possible explanation is that structural determinants such as stigma, social support, and the policy environment remained relatively stable, while countervailing factors (e.g., improvements in some health behaviors offset by deterioration in others) may have prevented widening disparities. It is also possible that existing health promotion efforts helped mitigate increases in disparities, although few interventions have been specifically designed for LGB+ populations in the UK. Although we observed a few statistically significant disparities in specific years, for instance, elevated harmful alcohol use among bisexual individuals in 2019–2020 and higher vaping prevalence among gay/lesbian individuals around 2019–2020, these differences did not represent consistent upward trends.

Notably, our results suggest that sexual minority groups are not uniformly at higher risk for substance use relative to heterosexual individuals, and that disparities—where they exist—tend to vary by substance type, sexual orientation subgroup, and sex. When interpreting these findings, it is also important to consider the wide confidence intervals around some estimates, especially in smaller subgroups, which limit the precision of year-specific comparisons and suggest caution in drawing strong conclusions based on isolated years.

One consistent and unexpected finding was that individuals identifying as “other” sexual minorities (including those who may be queer, pansexual, or asexual) exhibited substantially lower predicted probabilities of harmful alcohol drinking compared to heterosexual individuals across multiple years. This stands in contrast to prior research and theoretical expectations grounded in minority stress theory, which generally posit heightened risk among sexual minority populations due to social stigma and structural marginalization [9,10,15]. The reasons behind this inverse association are unclear but may relate to the heterogeneous composition of the “other” group or differences in cultural norms and social contexts within these communities. Further research is needed to understand the protective factors or behavioral profiles that might characterize this subgroup.

Our sex-stratified analyses revealed a few additional disparities, particularly among lesbian women. Smoking was more prevalent among lesbians in the early years of the study period (2015–2017), and they exhibited higher vaping prevalence compared to heterosexual women in 2019. These differences, although limited to certain years, align with some earlier studies reporting elevated substance use among sexual minority women, potentially driven by cumulative stress exposure, targeted marketing, or social drinking/smoking norms in lesbian communities [48]. Still, these disparities did not persist across the entire period and thus should be interpreted as potentially time-bound rather than steadily increasing.

We also found no significant differences in smoking between bisexual, gay/lesbian, and heterosexual individuals in most years. This finding diverges from earlier UK studies, including one using UKHLS data from 1991–2017, which reported consistently higher smoking rates among sexual minorities [26]. However, more recent work using repeated cross-sectional data from 2013–2019 found evidence of declining disparities, particularly for gay/lesbian individuals [27]. Our results are consistent with this latter finding, suggesting that smoking disparities between sexual minorities and heterosexuals have narrowed over time in the UK.

Regarding vaping, our study is among the first to use UK longitudinal data to assess sexual orientation–based disparities. We found some evidence of increased vaping among gay/lesbian individuals in 2019–2020, particularly among those aged 30 or above. This aligns with a recent cross-sectional study from the UK Millennium Cohort suggesting higher vaping rates among sexual minority youth, and with U.S.-based studies indicating higher vaping rates among adult gay men [49,50]. However, we did not find increased vaping among bisexual individuals, a result that mirrors previous findings from the English Smoking Toolkit Study [27].

Overall, the lack of substantial or widening disparities in substance use across most sexual minority groups from 2014 to 2021 may reflect either stable structural determinants (e.g., unchanged stigma, social support, policy environment) or countervailing trends (e.g., improvements in some health behaviors offset by deterioration in others). It is also possible that existing policies and health promotion efforts have mitigated potential increases in disparities, although few interventions have been evaluated specifically for LGBTQ+ populations in the UK. Our age-stratified analyses did not reveal substantial differences in substance use disparities between younger (under 30) and older (30 and over) sexual minority individuals. This suggests that, during the study period, the patterns of disparities were relatively consistent across age groups. Nonetheless, further research may be needed to explore how life stage transitions and related stressors—such as family formation, career development, or caregiving responsibilities—might interact with sexual orientation to influence substance use behaviors more subtly than what could be detected in our current dataset.

Our findings should be interpreted in light of several limitations. First, the use of self-reported measures for smoking, vaping, and alcohol use may be prone to social desirability bias, potentially underestimating true prevalence. Second, our binary classification of substance use behaviors (e.g., any vs. none) may obscure variation in intensity or frequency. Third, while we used weights and adjusted for major sociodemographic confounders, residual confounding may still exist. Finally, the wide confidence intervals—particularly among smaller subgroups—suggest caution when interpreting year-specific differences or subgroup comparisons. Despite these limitations, our study contributes new longitudinal evidence on trends in substance use disparities across a broad spectrum of sexual orientations using a nationally representative sample. The inclusion of sex- and age-stratified analyses further advances understanding of subgroup differences. Importantly, our findings suggest that concerns about sharply rising disparities may be overstated, although certain groups—particularly bisexual individuals and lesbian women—may remain vulnerable for specific types of substance use. Future work should prioritize larger samples of sexual minority subgroups, more precise measurement of gender identity and sexual orientation over time, and more nuanced behavioral outcomes. Given the unexpected lower risk among “other” sexual minorities, qualitative or mixed-methods research may help unpack the mechanisms underlying these patterns.

## Conclusion

Our study did not find clear or consistent patterns of increasing disparities in smoking, vaping, or harmful alcohol use across sexual orientation groups in the UK from 2014 to 2021. While we observed isolated differences—such as higher harmful alcohol use among bisexual individuals in some years and increased vaping among gay/lesbian individuals—these disparities did not appear to systematically widen over time. Conversely, the finding of consistently lower harmful drinking among “other” sexual minority individuals highlights the diversity of experiences within these communities and suggests a need for more nuanced research. Overall, our results underscore the importance of ongoing monitoring of substance use behaviors within sexual minority groups while cautioning against overgeneralizing disparities or assuming uniform vulnerability. Future research should examine the complex interplay of social stressors, policy environments, and community norms to identify effective, inclusive health interventions that recognize the heterogeneity of LGBTQ+ populations.

## Supporting information

S1 File Online Appendix. (PDF)
