# Supplementary material for: Longitudinal trends in vaping, smoking, and harmful alcohol use across sexual orientations in the UK (2014–2021)
Source: PLoS One. 2026 Jan 16;21(1):e0339847. doi: 10.1371/journal.pone.0339847 (PMC12810789; doi:10.1371/journal.pone.0339847)

Online Appendix

Table S1: Sample sizes across sexual orientations and years

Year	Heterosexual	Gay/lesbian	Bisexual	Other	Prefer not to say
Harmful alcohol drinking					
2015	17,463	287	307	219	484
2016	13,262	213	274	165	406
2017	18,108	317	370	238	492
2018	12,521	199	281	162	404
2019	15,967	266	308	200	403
2020	16,804	277	340	197	505
2021	23,444	406	458	270	620
Smoking					
2014	19,420	297	310	253	527
2015	32,656	520	576	415	995
2016	33,600	541	649	425	995
2017	31,695	518	620	413	931
2018	30,650	509	624	409	917
2019	28,553	468	569	374	821
2020	26,415	435	514	317	736
2021	23,744	410	464	277	640
Vaping					
2016	17,819	300	320	227	479
2017	30,192	495	580	389	871
2018	30,319	504	618	399	894
2019	28,269	464	561	365	797
2020	26,234	434	506	313	725
2021	23,616	410	462	272	627

Table S2: Alcohol Use Disorders Identification Test (AUDIT-C) scoring system

AUDIT-C Scoring System					
Question	0	1	2	3	4
How often do you have a drink containing alcohol?	Never	Monthly or less	Two to four times a month	Two to three times a week	Four or more times a week
How many units of alcohol do you drink on a typical day when you are drinking?	0 to 2	3 or 4	5 or 6	7 to 9	10 or more
How often have you had 6 or more units if female, or 8 or more if male, on a single occasion in the last year?	Never	Less than monthly	Monthly	Weekly	Daily or almost daily

Table S3: Predicted disparities in harmful alcohol drinking across sexual orientations and years, adjusted for age, ethnicity, marital status, region, urban/rural residence, education, employment status, and personal income (reference = heterosexual)

	dy/dx	std. err.	z	P>z	[95% conf.	interval]
Gay/Lesbian						
2015	0.047	0.052	0.910	0.364	-0.055	0.149
2016	0.081	0.048	1.700	0.089	-0.012	0.175
2017	0.076	0.049	1.560	0.118	-0.019	0.171
2018	0.018	0.050	0.360	0.720	-0.080	0.116
2019	0.067	0.050	1.340	0.179	-0.031	0.166
2020	-0.013	0.045	-0.290	0.768	-0.100	0.074
2021	0.039	0.043	0.890	0.374	-0.046	0.123
Bisexual						
2015	0.027	0.067	0.400	0.688	-0.104	0.158
2016	0.048	0.058	0.830	0.405	-0.065	0.161
2017	0.016	0.051	0.320	0.747	-0.084	0.116
2018	-0.001	0.057	-0.020	0.987	-0.113	0.112
2019	0.114	0.051	2.250	0.024	0.015	0.213
2020	0.135	0.047	2.890	0.004	0.044	0.226
2021	0.037	0.045	0.830	0.406	-0.051	0.125
Other						
2015	-0.223	0.064	-3.480	0.001	-0.349	-0.098
2016	-0.252	0.076	-3.330	0.001	-0.400	-0.104
2017	-0.200	0.067	-3.000	0.003	-0.330	-0.069
2018	-0.092	0.101	-0.910	0.363	-0.291	0.107
2019	-0.198	0.069	-2.870	0.004	-0.333	-0.063
2020	-0.119	0.077	-1.540	0.123	-0.270	0.032
2021	-0.101	0.073	-1.380	0.168	-0.244	0.042

Table S4: Predicted disparities in smoking across sexual orientations and years, adjusted for age, ethnicity, marital status, region, urban/rural residence, education, employment status, and personal income (reference = heterosexual)

	dy/dx	std. err.	z	P>z	[95% conf.	interval]
Gay/Lesbian						
2014	-0.010	0.033	-0.310	0.755	-0.076	0.055
2015	0.058	0.033	1.760	0.078	-0.007	0.122
2016	0.045	0.032	1.430	0.152	-0.017	0.108
2017	0.045	0.032	1.410	0.158	-0.018	0.108
2018	0.036	0.032	1.140	0.254	-0.026	0.099
2019	0.033	0.032	1.060	0.289	-0.028	0.095
2020	0.032	0.032	1.020	0.306	-0.030	0.094
2021	0.051	0.033	1.560	0.120	-0.013	0.115
Bisexual						
2014	0.008	0.038	0.210	0.837	-0.066	0.082
2015	-0.011	0.027	-0.410	0.683	-0.064	0.042
2016	0.025	0.028	0.880	0.377	-0.030	0.079
2017	0.062	0.030	2.080	0.038	0.004	0.120
2018	0.075	0.031	2.410	0.016	0.014	0.136
2019	0.037	0.029	1.290	0.198	-0.019	0.093
2020	0.021	0.028	0.750	0.452	-0.034	0.077
2021	0.037	0.028	1.310	0.191	-0.018	0.093
Other						
2014	0.051	0.057	0.900	0.368	-0.060	0.162
2015	0.004	0.041	0.090	0.928	-0.076	0.084
2016	0.008	0.041	0.190	0.852	-0.073	0.088
2017	-0.009	0.038	-0.240	0.808	-0.083	0.064
2018	0.034	0.042	0.820	0.411	-0.048	0.116
2019	-0.019	0.037	-0.510	0.607	-0.092	0.054
2020	-0.045	0.035	-1.290	0.198	-0.114	0.024
2021	-0.023	0.041	-0.560	0.574	-0.103	0.057

Table S5: Predicted disparities in vaping across sexual orientations and years, adjusted for age, ethnicity, marital status, region, urban/rural residence, education, employment status, and personal income (reference = heterosexual)

	dy/dx	std. err.	z	P>z	[95% conf.	interval]
Gay/Lesbian						
2016	0.041	0.025	1.620	0.104	-0.009	0.091
2017	0.012	0.019	0.640	0.521	-0.024	0.048
2018	0.039	0.022	1.740	0.082	-0.005	0.082
2019	0.056	0.025	2.240	0.025	0.007	0.105
2020	0.051	0.028	1.840	0.066	-0.003	0.106
2021	0.036	0.022	1.680	0.092	-0.006	0.078
Bisexual						
2016	-0.025	0.025	-1.000	0.316	-0.073	0.024
2017	-0.019	0.012	-1.520	0.129	-0.043	0.005
2018	-0.031	0.010	-3.060	0.002	-0.051	-0.011
2019	0.014	0.019	0.720	0.472	-0.024	0.052
2020	-0.013	0.012	-1.100	0.269	-0.037	0.010
2021	-0.002	0.015	-0.120	0.906	-0.030	0.027
Other						
2016	-0.041	0.012	-3.380	0.001	-0.065	-0.017
2017	-0.027	0.018	-1.540	0.125	-0.062	0.008
2018	-0.042	0.009	-4.660	0.000	-0.060	-0.025
2019	-0.030	0.013	-2.320	0.020	-0.056	-0.005
2020	-0.010	0.020	-0.510	0.612	-0.049	0.029
2021	-0.007	0.023	-0.290	0.775	-0.051	0.038

Figure S1: Longitudinal disparities (by age group) of harmful alcohol drinking, smoking, and vaping disparities across sexual orientations versus heterosexual people: adjusted probability estimates and 95% confidence intervals for the difference between each non-heterosexual group vs the heterosexual group over time.

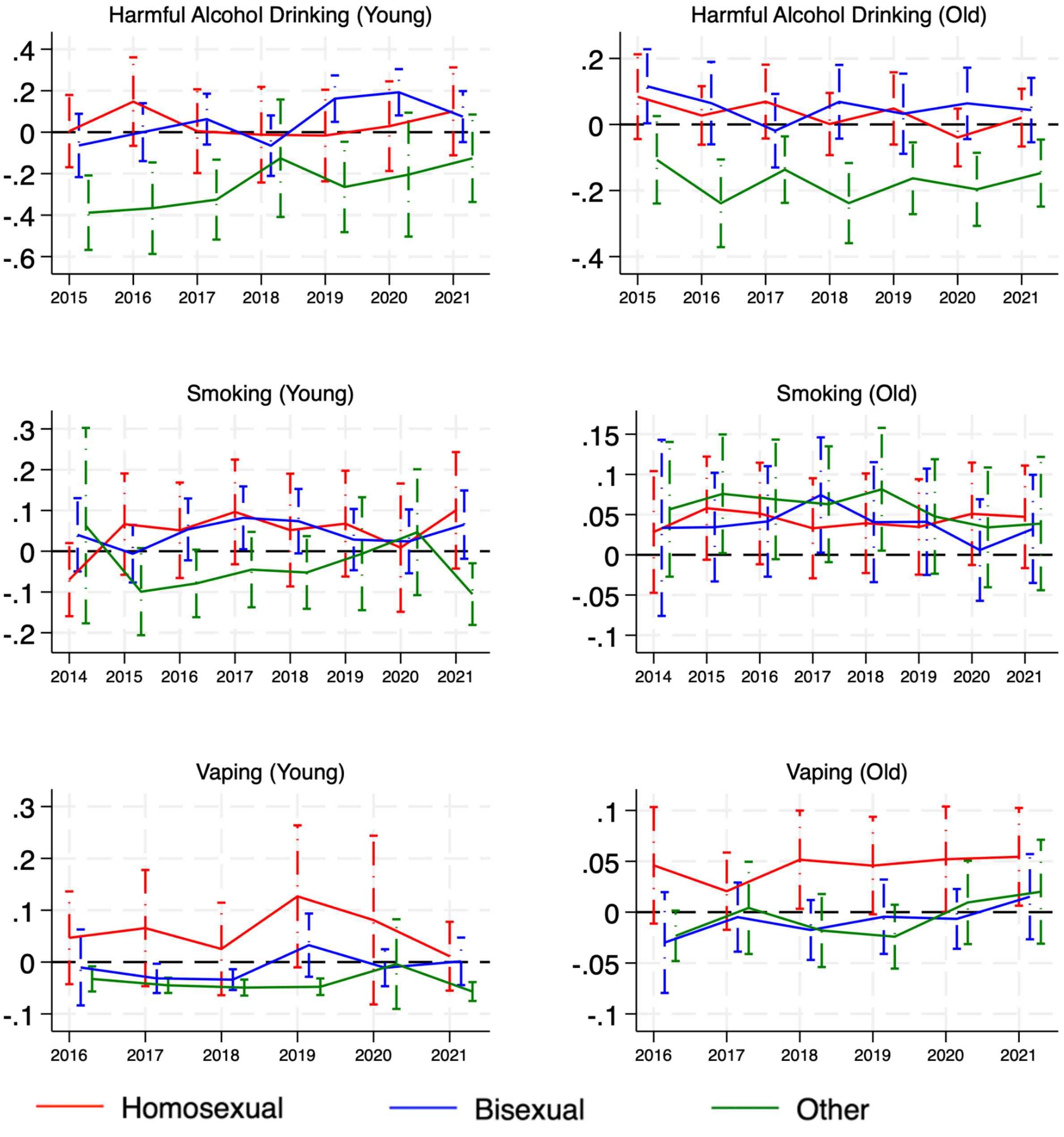

Figure S2: Longitudinal disparities (by partner status) of harmful alcohol drinking, smoking, and vaping disparities across sexual orientations versus heterosexual people: adjusted probability estimates and 95% confidence intervals for the difference between each non-heterosexual group vs the heterosexual group over time.

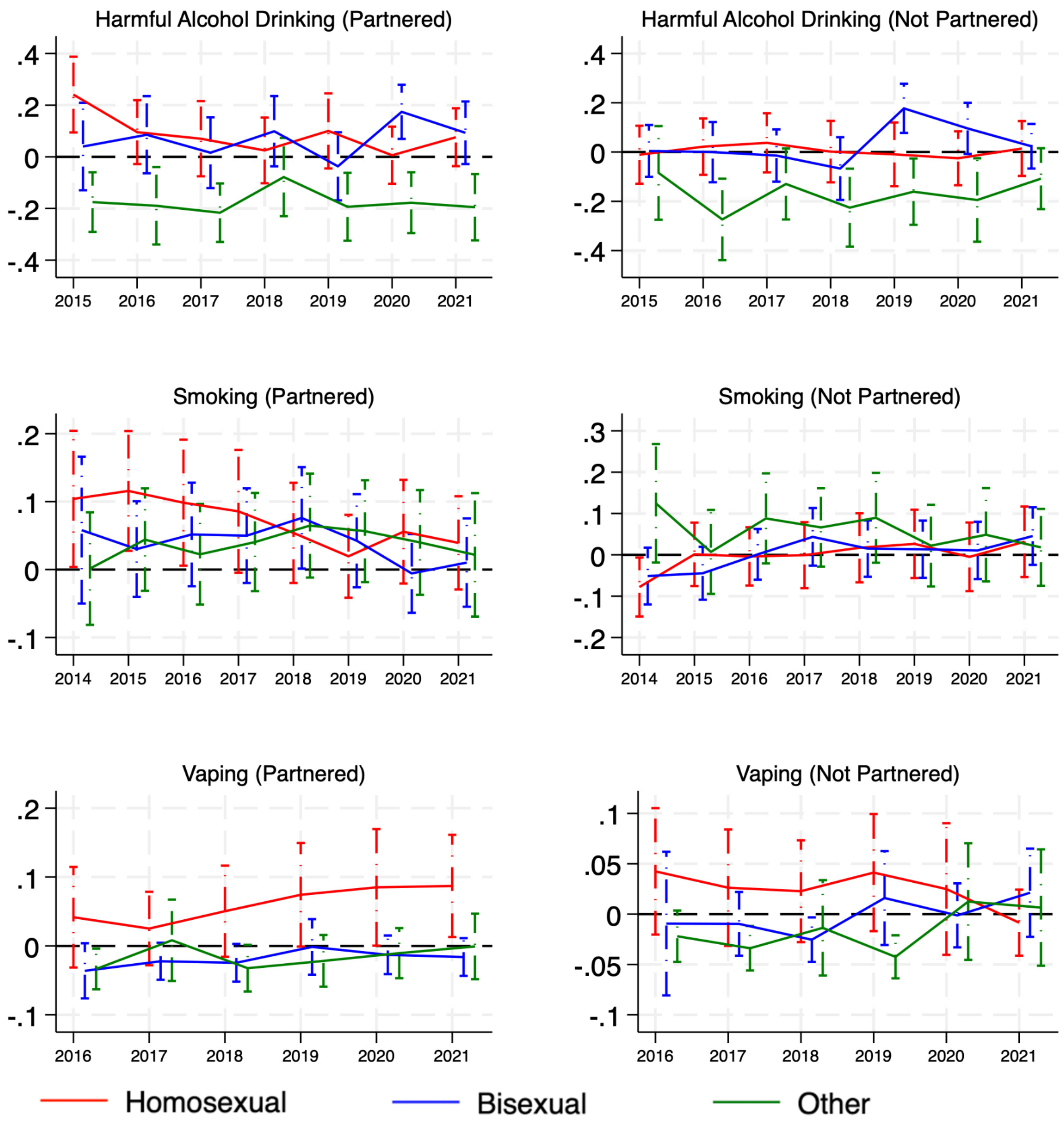

Figure S3: Longitudinal disparities (by parenthood status) of harmful alcohol drinking, smoking, and vaping disparities across sexual orientations versus heterosexual people: adjusted probability estimates and 95% confidence intervals for the difference between each non-heterosexual group vs the heterosexual group over time.

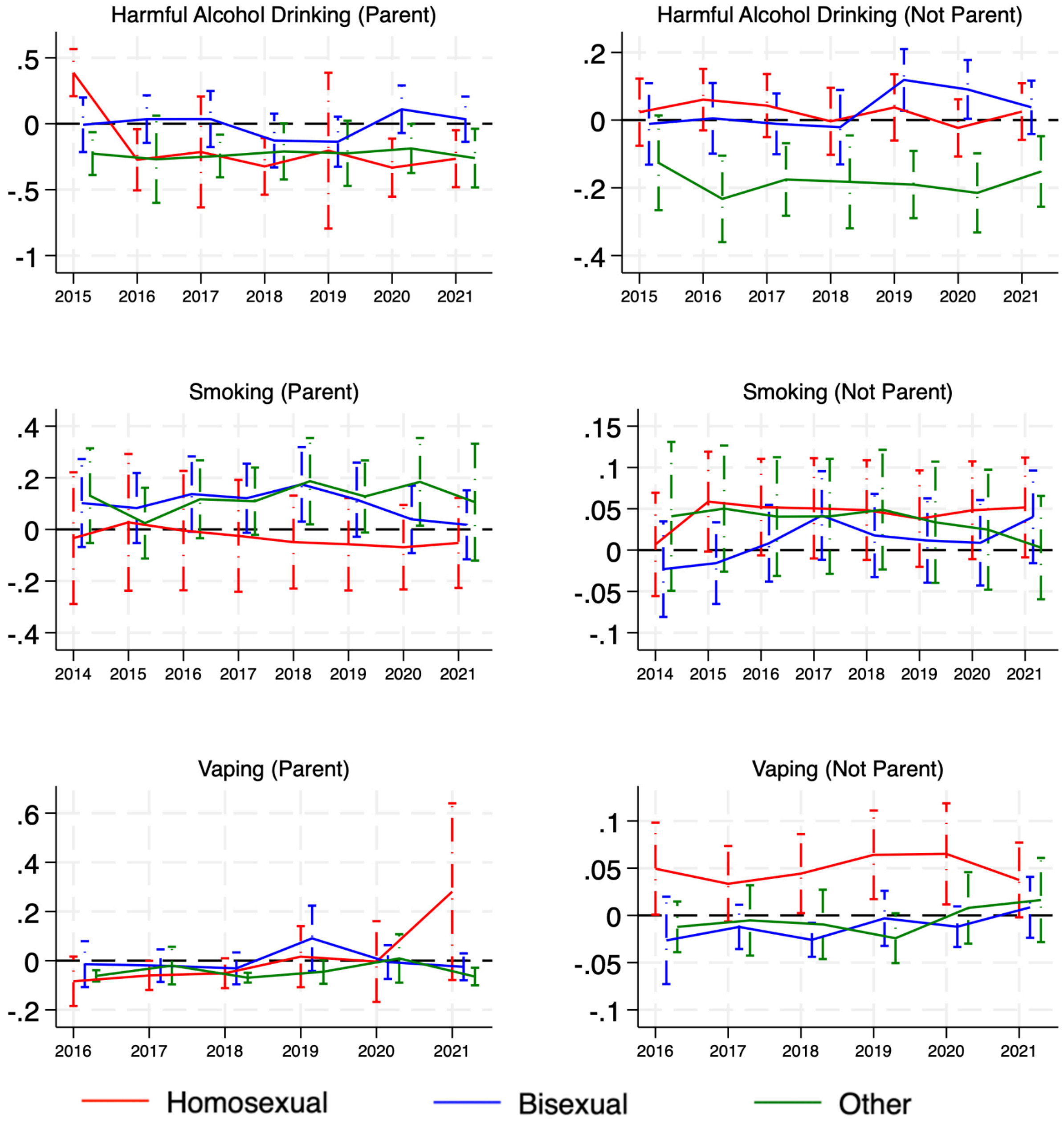

Figure S4: Longitudinal disparities of AUDIT-C score, number of cigarettes, and vaping (at least once a week) disparities across sexual orientations versus heterosexual people: adjusted estimates and 95% confidence intervals for the difference between each non-heterosexual group vs the heterosexual group over time.

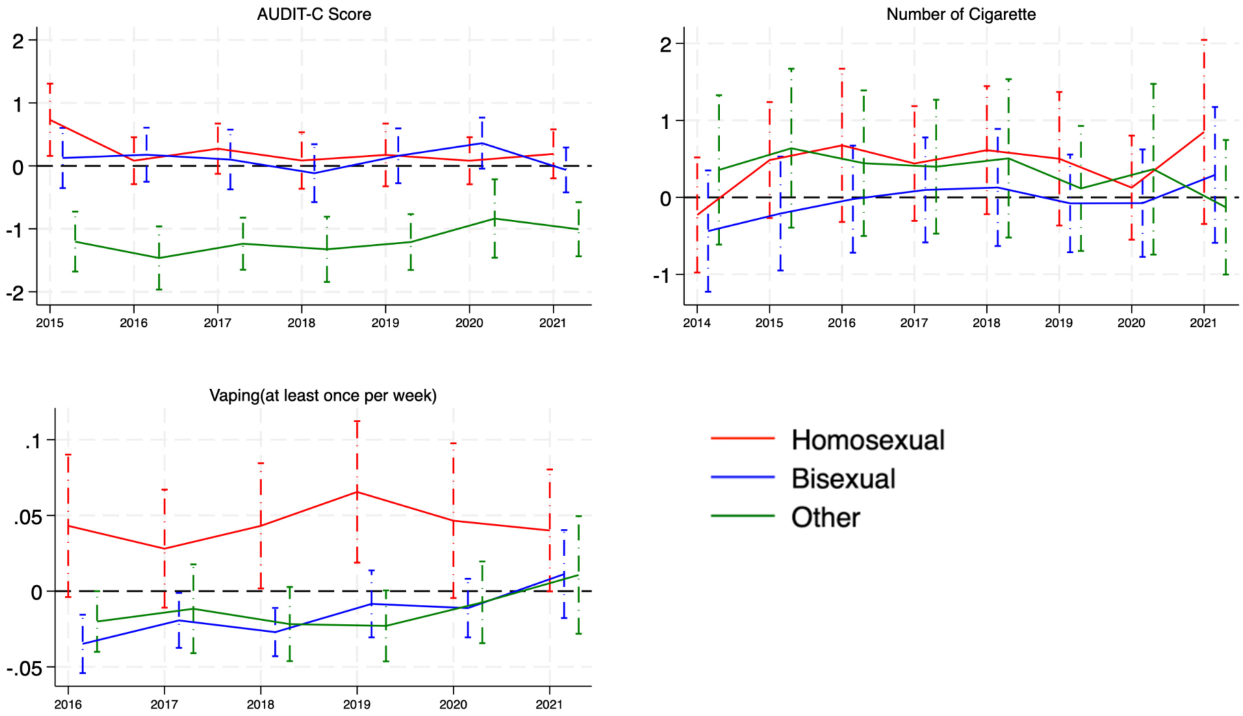

Supplement: S1 File — (PDF) [file pone.0339847.s001.pdf]
